# Meridionally consistent decline in the observed western boundary contribution to the Atlantic Meridional Overturning Circulation

**DOI:** 10.1126/sciadv.adz7738

**Published:** 2026-04-08

**Authors:** Qianjiang Xing, Shane Elipot, William E. Johns, David A. Smeed, Ben I. Moat, John W. Loder

**Affiliations:** ^1^ Rosenstiel School of Marine, Atmospheric, and Earth Science, University of Miami, Miami, USA.; ^2^ National Oceanography Centre, Southampton, UK.; ^3^ Bedford Institute of Oceanography, Fisheries and Oceans Canada, Dartmouth, Canada.

## Abstract

Despite numerous model-based analyses indicating a notable decline in the Atlantic Meridional Overturning Circulation (AMOC) in recent decades, robust, long-term evidence from multilatitudinal in situ observations remains limited. This study uses observational data from four mooring arrays, positioned along the western boundary of the North Atlantic (from 16.5°N to 42.5°N), to examine trends in the deep western overturning transports, derived from the cross-slope gradient in ocean bottom pressure or its equivalent, below and relative to 1000 meters that are linked to changing conditions at the western boundary. We identify a meridionally consistent decline in deep western overturning transport across these latitudes over the past two decades. This decline, observed at the western boundary, may serve as an effective indicator of AMOC weakening, despite the partial compensatory effect of overturn strengthening at the eastern boundary.

## Teaser

Western boundary observations in the Atlantic suggest a meridionally consistent decline in the ocean overturning circulation.

## INTRODUCTION

Climate models predict that the strength of the Atlantic Meridional Overturning Circulation (AMOC) should decrease with increasing greenhouse gas forcing (*1*–*3*). Model-based estimates and proxy reconstructions suggest that the AMOC is already in decline and could be at or near a critical tipping point, potentially leading to its collapse or shutdown (*2*–*8*). Continuous direct observations of the AMOC indicate a reduced state in recent years (*9*–*14*). However, these observations are relatively short compared to simulations and reconstructions, making it difficult to discern whether we are observing decadal-scale fluctuating variability or a declining trend of the AMOC consistent with expectations from climate change (*6*). Additional uncertainty in direct AMOC observations arises from their associated methodologies. The existing observational arrays, located at various latitudes within the Atlantic Ocean, have used different observation schemes and equipment, as well as methods of computation (*6*, *15*). Consequently, there is no consistent method for continuously estimating the strength of the AMOC across the Atlantic basin in modern times. Hence, we apply a common treatment to the now available observational data, to obtain consistent overturning transport estimates, which can then be used collectively to address research questions about the long-term variability of the AMOC.

Several studies (*16*–*19*) have proposed the use of measurements of the cross-slope (nominal zonal and down the ocean bottom) gradient of ocean bottom pressure (OBP) to estimate overturning transports. This method involves vertically integrating twice the OBP gradient at the eastern and western continental ocean boundaries within a range of chosen depths. The resulting difference between these two integrations is then used to obtain an overturning layer transport. A model study demonstrated that overturning transports derived solely from the OBP gradient at the western boundary can explain more than 90% of the interannual variability of the AMOC, as directly estimated from velocity fields (*16*). Furthermore, an observational study indicated that a western overturning transport, derived from the OBP gradient or its equivalent at the western boundary, can capture most of the total AMOC’s interannual variability at 26.5°N and 41°N (*19*). Therefore, a proposed observing strategy focuses on the western boundary contributions to overturning transports, assuming that changes in the OBP gradient at the eastern boundary are negligible or of secondary importance. Overturning transport anomalies in the North Atlantic, triggered by high-latitude mechanical and buoyancy forcing, potentially associated with variations in water mass formation rates, are carried southward at the western boundary by coastally trapped waves or current systems concentrated on the western boundary (*18*–*22*).

Building on this approach, additional research (*23*) generated four short-term (3.6 years) time series of western boundary contribution to the overturning transport relative to and below 1000 m derived from the western boundary OBP cross-slope gradient or its equivalent. These time series of overturning transports, clearly distinct from the transport of the Deep Western Boundary Current (DWBC) and hereafter referred to as deep western overturning transports, were obtained from four mooring arrays deployed at different latitudes within the North Atlantic (from 16.5°N to 42.5°N). The study revealed meridional coherence in these short-term overturning transport time series, primarily driven by wind stress (*23*). However, the limited duration of those time series precluded the assessment of long-term trends and decadal variability of the overturning signal at the western boundary.

In this study, we recompute four extended time series of the deep western overturning transports (see Materials and Methods). The observational data used are from four mooring arrays: the Meridional Overturning Variability Experiment (MOVE) array at 16.5°N (*11*), the Rapid Climate Change–Meridional Overturning Circulation and Heatflux Array (RAPID-MOCHA) at 26.5°N (*24*), the Woods Hole Oceanographic Institution Line W near 39.5°N (*25*), and the RAPID-Scotian line of the RAPID Western Atlantic Variability Experiment array near 42.5°N (*26*). These four overturning transport time series are denoted by their respective latitude of origin as *T*_16_, *T*_26_, *T*_39_, and *T*_42_. We emphasize that the four overturning transport time series are specifically designed to capture the variability of the AMOC as contributed by the western boundary only, rather than the variability due to both eastern and western boundaries. This focus is justified by both dynamical reasoning and observational constraints (*16*, *27*). We examine the long-term trends of these overturning transports and their relationship with the AMOC, aiming to address whether the apparent recent decline in the AMOC (*10*, *13*, *14*) is statistically significant and meridionally consistent.

## RESULTS

### General decline of deep western overturning transport

**Fig. 1.**
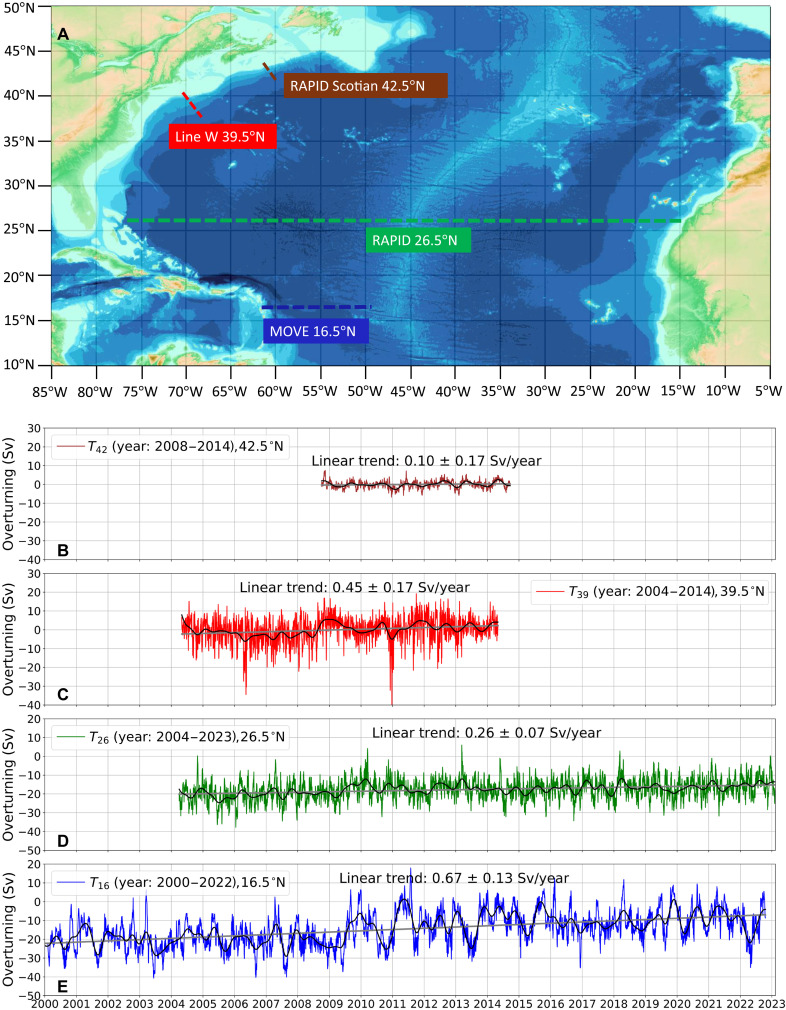
Deep western overturning transports in the North Atlantic. (**A**) General Bathymetric Chart of the Oceans (GEBCO) bathymetry (*50*) of the North Atlantic basin between 10°N to 50°N. The colored dashed lines depict the locations of the four observation arrays used in this study (RAPID-Scotian line, Line W, RAPID-MOCHA, and MOVE array). Time series of deep western overturning transport at RAPID-Scotian line (**B**), Line W (**C**), RAPID-MOCHA (**D**), and MOVE array (**E**). Note here that *T*_16_ and *T*_26_ are absolute transports, while *T*_39_ and *T*_42_ are transport anomalies. In each panel, the colored line is the daily time series, and the black solid line is a 3-month third-order Butterworth low-pass filtered version. The estimated uncertainties on the daily time series are 1.4, 1.5, 4.6, and 1.2 Sv, respectively from *T*_16_ to *T*_42_. The gray lines indicate the linear trend estimated for each daily time series by modeling mixed fixed and random effects (see Materials and Methods). Trend uncertainties correspond to 2-σ confidence intervals.

We observe a meridionally consistent, positive linear trend in the deep western overturning transports across the subtropical Atlantic basin, from 16.5°N to 42.5°N (Fig. 1). Note that we estimate the deep western overturning transport within the 1000- to 4000-m depth range, where negative values in the transport time series correspond to southward flow. Accordingly, a positive linear trend indicates a decline in overturning transport. The largest decline in deep western overturning transport is observed at the MOVE array (16.5°N), where *T*_16_ decreases at a statistically significant rate of 0.67 ± 0.13 Sv/year between 2000 and 2022 (reported uncertainties are 2-σ confidence intervals). This pronounced positive trend contrasts sharply with the slightly negative trend (a strengthening) in the estimated volume transport in the North Atlantic Deep Water (NADW) layer (1200 to 4950 m) at the same MOVE array when calculated following the method in (*11*). We discuss the reasons behind this discrepancy in the subsequent section. At 26.5°N, *T*_26_ shows a significant declining trend of 0.26 ± 0.07 Sv/year between 2004 and 2023, which is larger than the decline in AMOC transport estimated by the RAPID-MOCHA array (*24*). At Line W (39.5°N), *T*_39_ also exhibits a significant declining trend of 0.45 ± 0.17 Sv/year over the period 2004–2014. This trend also surpasses the reported decline for the same period of the AMOC transport at 35°N (*9*), estimated by integrating data from Line W, Argo floats (*28*), and satellite altimeter observations at nearby latitudes. At the RAPID-Scotian line (42.5°N), *T*_42_ exhibits a declining trend of 0.10 ± 0.17 Sv/year between 2008 and 2014, which is not statistically significant but is consistent with the trends observed at the other three latitudes. When the trends of these four times series are re-estimated over various overlapping time periods, they are consistently found to be positive and significant and increasing in magnitude from high to low latitudes (except in one instance; table S1).

Model studies indicate a declining trend in AMOC over the recent decades (*2**,* *29*). The multimodel ensemble mean of the AMOC anomaly at 35°N from the Coupled Model Intercomparison Project Phase 6 reflects a decline at a rate of approximately 7.6 Sv per century during 1985–2014 (*2*). Similarly, nearly all Ocean Model Intercomparison Project Phase 2 simulations suggest an AMOC weakening at 26.5°N over 2004–2018 at a mean decline rate of 0.15 ± 0.16 Sv/year [see figure 19 in (*29*)]. In summary, the simulated AMOC also shows a decline during the historical period, however with a linear trend that is weaker than our estimated deep western overturning transports.

### Deep western overturning transport versus AMOC

We assess whether the deep western overturning transport below and relative to 1000 m, estimated from western boundary arrays (*17*, *23*), reliably represents the long-term trend and variability of the AMOC and to what extent the deep western overturning transport could be considered a method for monitoring the AMOC. We focus on the results at 26.5°N, where the longest continuous observational estimates of AMOC strength have been published from the RAPID-MOCHA program (*24*), and at 16.5°N, where long-term estimates of the deep limb of the AMOC have been derived from the MOVE array (*11*).

**Fig. 2.**
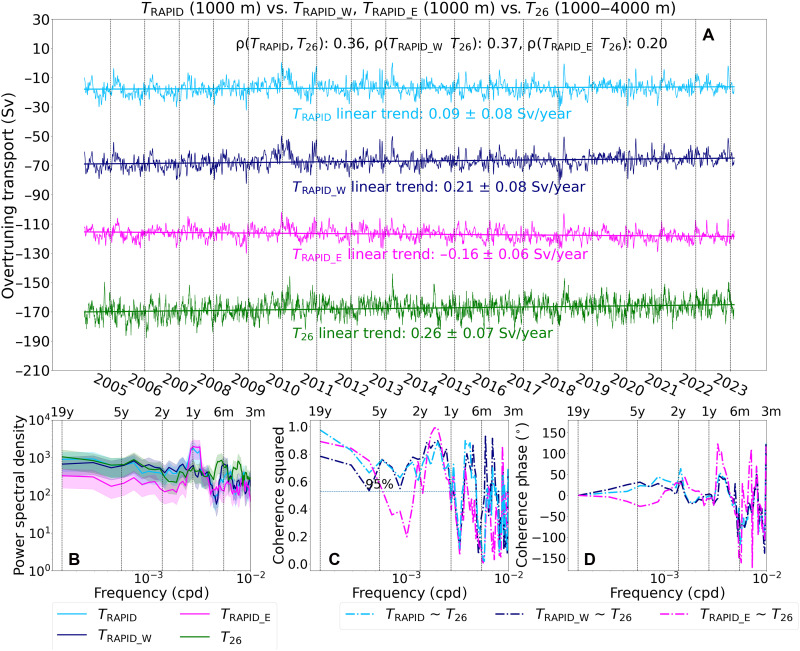
Comparisons between AMOC estimates and deep western overturning transport estimates at the RAPID-MOCHA array. (**A**) Time series of *T*_RAPID_, *T*_RAPID_W_, *T*_RAPID_E_, and *T*_26_ during April 2004 to February 2023, successively offset by −50 Sv. Note that *T*_RAPID_, *T*_RAPID_W_, and *T*_RAPID_E_ are derived from the estimated official AMOC transport by changing their signs to negative for the convenience of the comparison with *T*_26_. (**B**) Multitaper spectra (*49*) of *T*_RAPID_, *T*_RAPID_W_, *T*_RAPID_E_, and *T*_26_ with their linear trends removed (y, year; m, month; cpd, cycle per day). Colored shades indicate the 95% confidence level for the power spectrum. (**C**) Multitaper coherence squared and (**D**) coherence phase between *T*_RAPID_ and *T*_26_, between *T*_RAPID_W_ and *T*_26_, and between *T*_RAPID_E_ and *T*_26_. The horizontal blue dashed line in (C) indicates the 95% confidence level for coherence squared. Note that the correlations, $\rho \left(T_{\text{RAPID}},T_{26}\right)$, $\rho \left(T_{\text{RAPID}\_W},T_{26}\right)$, and $\rho \left(T_{\text{RAPID}\_E},T_{26}\right)$ are calculated on the basis of the detrended overturning transport time series. All *P* values for correlations are significantly smaller than 0.05.

A previous study conducted comparisons between *T*_26_ and the time series of the maximum of the overturning streamfunction (AMOC), hereafter *T*_RAPID_, derived from the RAPID-MOCHA array over a 12-year period (2004–2011) (*19*). They found that *T*_26_ effectively captured linearly the interannual variability of *T*_RAPID_ (*19*) but did not investigate long-term trends. The latest update of *T*_RAPID_ exhibits a significant decline of 0.09 ± 0.08 Sv/year from 2004 to 2023 (Fig. 2A), which is almost three times weaker than the declining trend of *T*_26_ at 0.26 ± 0.07 Sv/year for the same period. To investigate the difference in the trends between *T*_26_ and *T*_RAPID_, we isolate the western or eastern boundary contribution to *T*_RAPID_. We recalculate the overturning streamfunction and its maximum by holding the eastern boundary dynamic height fixed at its time-mean value during 2004–2023, resulting in a new time series here called *T*_RAPID_W_ (*19*, *27*). This time series exhibits a stronger and significant decline of 0.21 ± 0.08 Sv/year, consistent within error bars with the trend of *T*_26_ (Fig. 2A). Conversely, by holding constant at time-mean value for all contributions to the transport calculation at the western boundary, including the Florida Straits transport, the western wedge, and the western boundary dynamic height (*30*), we obtain the eastern boundary contribution to the AMOC at 26.5°N, *T*_RAPID_E_, which exhibits a significant strengthening (negative) trend of −0.16 ± 0.06 Sv/year (Fig. 2A). Considering that the Ekman transport contribution to the AMOC at 26.5°N does not exhibit a significant trend from 2004 to 2023 (−0.03 ± 0.06 Sv/year), these results suggest that the western and eastern boundary contributions to the AMOC are responsible for the trend signals but act in opposing directions. Note that the trend in the *T*_RAPID_ cannot be obtained by simply summing the trends in *T*_RAPID_W_ and *T*_RAPID_E_. This is because *T*_RAPID_, *T*_RAPID_W_, and *T*_RAPID_E_ all include the varying Ekman transport. Consequently, summing the trends in *T*_RAPID_W_ and *T*_RAPID_E_ would result in double-counting the Ekman transport trend. Thus, the deep western overturning transport estimate at 26.5°N (*T*_26_) captures the sign of the AMOC trend but overestimates the size of the trend due to compensating effects of the eastern boundary on the total AMOC.

After removing their linear trends, *T*_RAPID_ and *T*_RAPID_W_ show similar correlations with *T*_26_, at 0.36 and 0.37, respectively, indicating that *T*_26_ captures little of the total variance of the AMOC. However, the three time series (*T*_RAPID_, *T*_RAPID_W_, and *T*_26_) all display similar power spectrum at low frequencies (Fig. 2B), and the coherence squared between both *T*_RAPID_ and *T*_RAPID_W_ and *T*_26_ is significantly high, with coherence phases approaching zero at low frequencies (Fig. 2, C and D). In conclusion, *T*_26_ is well suited to capture linearly the interannual and decadal variability of the AMOC at 26.5°N. *T*_26_ does not, however, accurately capture the linear trend of the AMOC since it can only account for changes at the western boundary, and changes at the eastern boundary appear to have significantly affected the total AMOC trend at 26.5°N over the RAPID observational period.

**Fig. 3.**
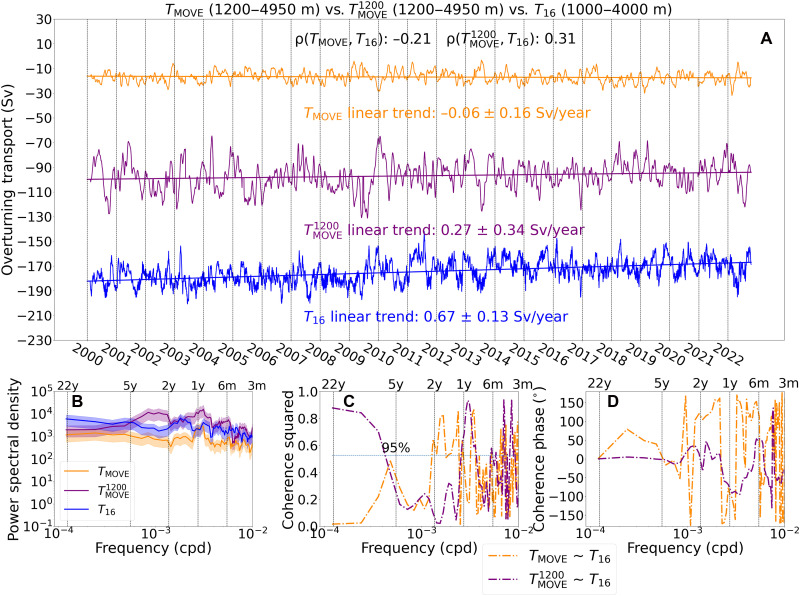
Comparisons between the NADW layer transport estimates and deep western overturning transport estimates at the MOVE array. (**A**) Time series of *T*_MOVE_, $T_{\text{MOVE}}^{1200}$, and *T*_16_ during January 2000 to October 2022, successively offset by −70 Sv. (**B**) Multitaper spectra of *T*_MOVE_, $T_{\text{MOVE}}^{1200}$, and *T*_16_ with their linear trends removed. Colored shades indicate the 95% confidence level for the power spectrum. (**C**) Multitaper coherence squared and (**D**) coherence phase between *T*_MOVE_ and *T*_16_; between $T_{\text{MOVE}}^{1200}$ and *T*_16_. The correlations, $\rho \left(T_{\text{MOVE}},T_{16}\right)$ and $\rho \left(T_{\text{MOVE}}^{1200},T_{16}\right)$, are calculated on the basis of the detrended overturning transport time series. All *P* values are estimated to be significantly smaller than 0.05.

The MOVE array is designed to monitor the deep limb of the AMOC by measuring the southward transport of NADW, hereafter *T*_MOVE_, across the western portion of the Atlantic basin at 16.5°N, from the arc of the Antilles (effectively the western boundary) to the Mid-Atlantic Ridge (*11*). *T*_MOVE_ shows an insignificant strengthening trend from 2000 to 2022 (−0.06 ± 0.16 Sv/year; Fig. 3A), in sharp contrast to *T*_16_, which exhibits the strongest declining trend (0.67 ± 0.13 Sv/year) over the same period. *T*_16_ shows a generally higher power spectrum than *T*_MOVE_, meaning that *T*_16_ exhibits higher variance across all frequencies (Fig. 3B). The detrended correlation between *T*_MOVE_ and *T*_16_ is weakly negative (−0.21), with low coherence squared at low frequencies (Fig. 3C). A primary difference in deriving *T*_MOVE_ and *T*_16_ is the choice of reference level: *T*_MOVE_ uses a no-motion reference level of 4950 m, which is the assumed time-mean interface between southward-flowing NADW and northward-flowing Antarctic Bottom Water, while *T*_16_ uses 1000 m, the assumed time-average depth of the maximum of the overturning streamfunction throughout the subtropical region of the North Atlantic (*3**,* *31**,* *32*). To assess the impact of this reference level difference, we recalculate the southward transport of NADW at the MOVE array data but using instead an upper reference level of 1200 m. This depth level corresponds to the top-most level of direct velocity measurements along the western boundary for the array. With this adjustment, the new time series, called hereafter $T_{\text{MOVE}}^{1200}$, shows an insignificant declining trend of 0.27 ± 0.34 Sv/year from 2000 to 2022 (Fig. 3A), with power spectrum similar to that of *T*_16_ across all frequencies (Fig. 3B). In addition, $T_{\text{MOVE}}^{1200}$ and *T*_16_ exhibit a positive correlation (0.31) and significantly high (more than 0.8) coherence squared on decadal timescales. These results imply that changing the reference level brings into agreement $T_{\text{MOVE}}^{1200}$ and *T*_16_ in terms of both their long-term trends and overall variability. Another distinction between *T*_MOVE_ and *T*_16_ is that their integration limits differ. To assess the impact of this difference, we recalculate the deep western overturning transport by integrating from 1200 to 4950 m, using 1200 m as the reference level. The resulting time series exhibits a similar temporal evolution to *T*_16_ but shows a stronger declining trend of 0.74 ± 0.19 Sv/year (fig. S1A). This suggests that integration limits affect only the magnitude of trend of the deep western overturning transport, without altering its sign.

Similar to the difference between *T*_RAPID_ and *T*_26_, our derivation of *T*_16_ also differs conceptually from that of *T*_MOVE_ in capturing different boundary contributions. *T*_MOVE_ quantifies the transport of the NADW layer from the western boundary to the Mid-Atlantic Ridge, whereas *T*_16_ represents the deep western overturning transport based solely on the equivalent of the OBP gradient at the western boundary of the Atlantic basin. We recalculate both *T*_MOVE_ and $T_{\text{MOVE}}^{1200}$ by holding their eastern (Mid-Atlantic Ridge) dynamic height or western (Antilles) dynamic height and western wedge transport as constant over time. The resulting time series (fig. S1, B to E) reveal that the western and eastern boundary contributions to the NADW transport at 16.5°N are responsible for opposing trend signals. This pattern is consistent with the opposing influences of the western and eastern boundary contributions to the AMOC at 26.5°N. Specifically, the linear trend of $T_{\text{MOVE}\_W}^{1200}$ (0.52 ± 0.30 Sv/year) is closer to that of *T*_16_ compared to the trend of $T_{\text{MOVE}}^{1200}$. On the basis of the findings above, we conclude that the substantial difference from the trend of *T*_16_ to the one of *T*_MOVE_ stems from two primary factors: the exclusive focus on western boundary variability and the use of an upper reference level (*33**,* *34*). Despite this discrepancy observed at 16.5°N, we observe that the deep western overturning transport effectively captures the interannual variability of the AMOC and is able to capture its long-term trend signal at the western boundary at 26.5°N (see above). Given these results, we argue that the calculation of deep western overturning transport should be consistently applied at four latitudes across the North Atlantic to ensure the methodological consistency of this study.

### The impact of depth limits

All four overturning transport time series in this study are calculated consistently, over a depth-limited layer, spanning from an upper reference depth (1000 m) to a lower integration depth (~4000 m). The calculation assumes a fixed reference level at 1000 m, corresponding to the assumed mean depth of the maximum of the overturning streamfunction (fig. S2B). However, the RAPID-MOCHA observations reveal that this depth varies between ~600 and 1200 m and that it has shoaled at a rate of 1.7 m/year between 2004 and 2023 (fig. S3) (*24*). The lower integration depth is defined by the zero crossing of the overturning streamfunction, which marks the transition between upper overturning cell and lower overturning cell of the 26.5°N section (see the schematic of two overturning cells in fig. S2A) (*35*). The RAPID-MOCHA data indicate that this lower integration depth exhibits temporal variability, ranging between 3500 and 4800 m (fig. S2B) (*24*).

**Fig. 4.**
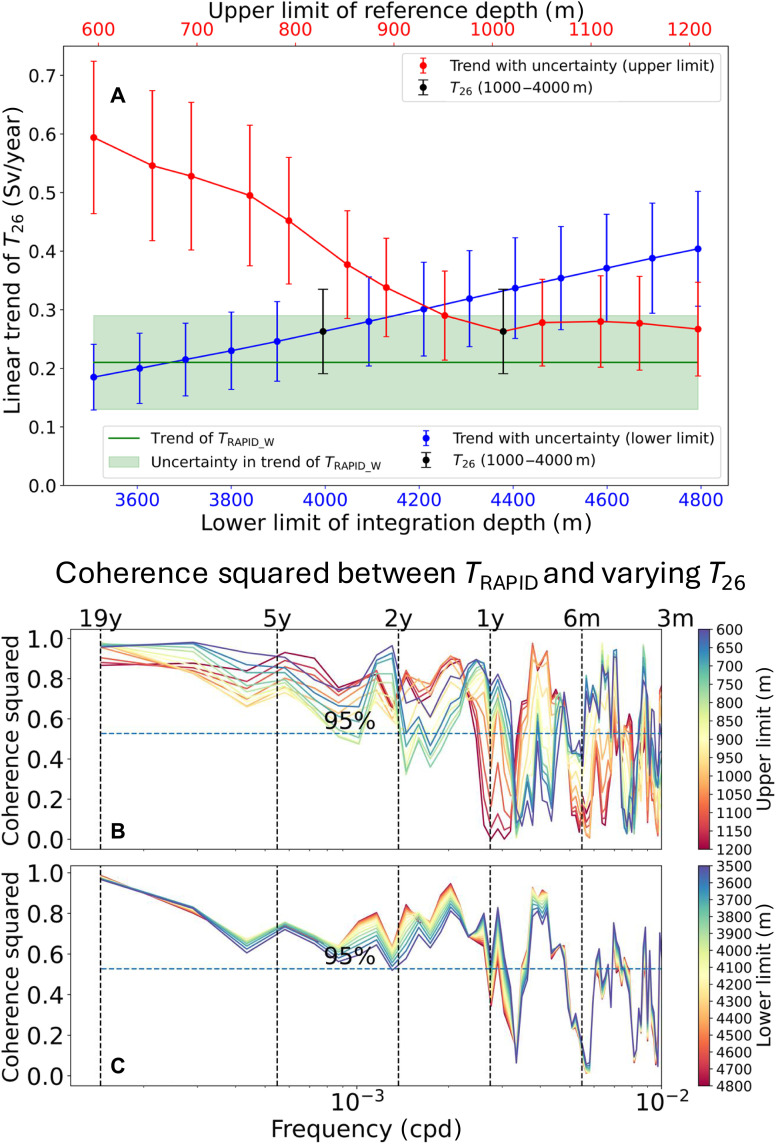
Sensitivity experiments of varying *T*_26_ to upper limit of reference depth and lower limit of integration depth. (**A**) Sensitivity of linear trend of varying *T*_26_. The upper reference depth (red) varies incrementally from 1200 to 600 m, and the lower integration depth (blue) varies from 3500 down to 4800 m. Error bar of every point shows the uncertainty of 2-σ confidence intervals for the linear trend. Black point and error bar shows the control case of the *T*_26_ with an integration layer of 1000 to 4000 m. Green horizontal line shows the trend of *T*_RAPID_W_, and the surrounding shade indicates its associated uncertainty. (**B**) Coherence squared between *T*_RAPID_ and varying *T*_26_ with a different upper limit of reference depth (in meters). (**C**) Coherence squared between *T*_RAPID_ and varying *T*_26_ with a different lower limit of integration depth (in meters).

To investigate the impacts of fixed upper and lower depth limits on estimating the trend of the deep western overturning transport, we conduct some sensitivity experiments with the data from the RAPID-MOCHA array. When the fixed upper limit is lowered from 1000 to 1200 m, the linear trend of *T*_26_ does not significantly change (Fig. 4A). In contrast, when the upper limit is raised from 1000 to 600 m, the positive trend of *T*_26_ intensifies significantly, reaching 0.59 ± 0.13 Sv/year, with a corresponding increase in trend uncertainty. When the lower limit depth is changed from 3500 to 4800 m, the positive trend of *T*_26_ grows markedly, rising to 0.40 ± 0.10 Sv/year. Beyond the trend, the coherence squared estimates between *T*_RAPID_ and each of the *T*_26_ variants (Fig. 4, B and C) indicate that their linear relationship is generally unaffected by changes in either the upper or lower depth limits.

To account for the potential influence of a linear temporal change of the upper reference depth, we allow this one to linearly decrease from 1000 to 970 m in our calculation of *T*_26_, accordingly to the observations of the AMOC streamfunction maximum over the 2004–2023 time period (fig. S3). This changing reference depth leads to an approximate 15% reduction of the trend for *T*_26_, to 0.22 ± 0.07 Sv/year, bringing it closer to the trend of *T*_RAPID_W_, which takes into account, by construction, the time-varying depth of the overturning streamfunction maximum.

The sensitivity tests conducted above collectively suggest that upper or lower depth limits of integration primarily influence the magnitude, but not the sign, of the long-term linear trend of the deep western overturning transport and have little effect on how much they linearly capture the interannual variability of the AMOC. This also confirms the viability of using a deep western overturning transport, referenced to a fixed 1000-m level, to capture the trend signal of the AMOC emanating from the western boundary.

## DISCUSSION

The methods and technologies used to measure the AMOC at various locations in the North Atlantic vary depending on the characteristics of the local circulation, the logistical constraints of observations, and the objectives of individual monitoring programs (*15*). Thus, maintaining a consistent methodology for continuously and accurately monitoring the AMOC across various latitudes of the Atlantic basin presents a considerable challenge for the physical oceanography community. As proposed by Hughes *et al.* (*36*), long-term observations of OBP is a promising approach for monitoring large-scale ocean circulation, as they provide high accuracy while minimizing the influence of mesoscale variability. Our results further indicate that the estimation of the deep western overturning transport, derived from the western OBP gradient or equivalently the western geostrophic transport shear, can act as a reasonable method to capture interannual variability of the AMOC and indicate trends related to signals occurring at the western boundary. In measurements at four locations in the low-to-mid latitudes of the North Atlantic, we reveal a meridionally consistent declining trend in the deep western overturning transport which is statistically significant within two SEs at three latitudes (16.5°N, 26.5°N, and 39.5°N).

Our study relies on mooring observations at the western boundary only. Only at the RAPID-MOCHA array can they be compared to a fully constrained AMOC estimate that includes variability at the eastern boundary. The western boundary of the North Atlantic is the boundary of action for the AMOC, where dynamical variability is more pronounced compared to the eastern boundary (*27**,* *37*), a classical feature of the general oceanic circulation. In addition, it is the boundary where signals from high latitudes are expected to manifest themselves first. In terms of long-term trend, we find that while the signal from the western boundary is one of decline, the signal from the eastern boundary is one of strengthening, yet one that does not entirely balance the western boundary. In a sense, observations at the western boundary, in isolation from the eastern boundary, constitute the canary in a coal mine for the tendency of the AMOC. Arguably for this reason, the transport of the DWBC in the North Atlantic has been considered a proxy for AMOC variability (*25**,* *38**–**40*). However, DWBC transport and deep western overturning transport are markedly different ways of capturing overturning transports (*18*). The DWBC transport at 26.5°N has no correlation with the AMOC at that latitude (*41*). In contrast, the deep western overturning transport derived here has been shown to relate linearly to the AMOC. Thus, signals from deep western overturning transport may arguably serve as an effective indicator of potential AMOC changes, even if they are partially offset by signals at the eastern boundary, as exemplified by the observed linear trends at 26.5°N and 16.5°N (Fig. 2A and fig. S1). Because of limitations in the current observational data, it remains unclear whether the opposing trend pattern between the western and eastern boundary overturning is consistent over the entire North Atlantic, and what dynamical mechanisms underlie this contrast. These questions warrant further investigation using ocean models.

Regarding our primary research questions, we find that deep western overturning transport estimates have been undergoing a notable meridionally consistent decline over the past two decades, but questions remain regarding the role of contributions at the eastern boundary in terms of mitigating the declining signal from the west. Our findings propose that deep western overturning transports, below and relative to 1000 m, could be monitored continuously throughout the Atlantic basin, as this approach shows promise as a candidate for future AMOC observing systems. Yet, continued monitoring of the eastern boundary also appears essential to fully capture the long-term trend of the AMOC and to investigate the underlying dynamics.

## MATERIALS AND METHODS

### Measuring zonally integrated meridional transports

Measuring meridional transports starts by considering the zonal component of the horizontal geostrophic momentum balance

$$\rho v_{g}=\frac{1}{f}\frac{\partial p}{\partial x}$$ (1)

where ρ is the seawater density, *v*_g_ is the meridional geostrophic velocity, *f* is the Coriolis parameter, and *p* is the pressure. The coordinates (*x*, *y*, and *z*) are in the directions (east, north, and up), respectively. By taking the zonal integral of this equation between the western and eastern boundaries of an ocean basin, we define *M*(*y*, *z*), the geostrophic meridional mass transport per unit depth at latitude *y*

$$M\left(y,z\right)\equiv \int_{x_{W}}^{x_{E}}\rho v_{g}\;\mathrm{dx}=\frac{1}{f}\left[p_{E}\left(y,z\right)-p_{W}\left(y,z\right)\right]$$ (2)

This expression states that *M*(*y*, *z*) is the difference of pressures on the eastern and western boundaries, respectively located at zonal positions *x*_W_ and *x*_E_. Because the physical boundaries of an ocean basin are typically continental slopes, the ocean pressure of interest is in practice the OBP across these slopes.

Rather than depth-independent transports, our interest lies in overturning transports, that is, transports of ocean mass that exhibit vertical shear and are associated with compensating flows at different depths (*16*). Hence, it is not so much absolute OBP which is of interest to determine transports but rather its vertical gradient. By taking the vertical derivative of (*2*), we simply obtain

$$\frac{\partial }{\partial z}M\left(y,z\right)=\frac{1}{f}\frac{\partial }{\partial z}\left[p_{E}\left(y,z\right)-p_{W}(y,z)\right]$$ (3)

This expression can be used to state that the total *z*-dependent overturning transport can be traced back to two separable contributions: one from the OBP gradient at the eastern boundary from the first term on the right-hand side and one from the OBP gradient at western boundary from the second term of the same side. As explained in (*17*), each of these two terms can be integrated to obtain transports representing overturnings, inasmuch as constants of integration are chosen to ensure a zero net top-to-bottom meridional transport in each case.

In an idealized ocean with vertical sidewalls, the vertical pressure gradient is proportional to density through the hydrostatic relation, ∂p/∂z=−ρg with g acceleration of gravity, so that Eq. 3 becomes

$$\frac{\partial }{\partial z}M\left(y,z\right)=\frac{g}{f}\left[\rho_{W}\left(z\right)-\rho_{E}\left(z\right)\right]$$ (4)

This new expression is actually the underpinning for estimating oceanic mass transports between pairs of dynamic height moorings separated by an arbitrary zonal distance (*42*). In reality, the ocean does not exhibit vertical sidewalls but sloping boundaries. Hence, there exist two observational approaches to estimating meridional transports over an entire ocean basin. The first approach consists in dividing a basin in two types of regions: a vertical-sidewalled rectangular section delineated by dynamic height moorings where Eq. 4 can be applied and roughly triangular regions to the west and east of the rectangular section where ocean velocities are measured directly by current meters. This is partly the approach taken at the RAPID-MOCHA and MOVE arrays for which transports within western wedges are directly measured with current meters, and interior transports further east are measured with dynamic height moorings (*11**,* *30*).

As demonstrated in (*17*), the second approach that can be taken in the presence of sloping boundaries is to generalize the hydrostatic equation by combining it with the geostrophic relationship to obtain an expression for the gradient of OBP (*p*_b_) at the slopes as

$$\frac{\partial p_{b}}{\partial z}=-\frac{\rho {\mathrm{fV}}_{L}}{H_{s}}-\rho g$$ (5)

where *V*_L_ is an along-slope velocity directed to the left of a three-dimensional path along the sloping boundary, and *H*_s_ is the slope magnitude of the path. This expression can be substituted in Eq. 3 for the vertical gradient of the pressure on the western boundary, or on the eastern boundary, or both, to obtain a western overturning transport, or an eastern overturning transport, or a total overturning transport. In (*17*), specific procedures are outlined, and in situ tests are detailed, demonstrating how to apply this expression using oceanic velocity and density measurements acquired in discrete steps along continental slopes. In particular, the in situ absolute density is used for the first term on the right hand side of Eq. 5, but the density anomaly with respect to a reference density profile is used in the second term [equation 13 in (*17*)]. This “stepping method” has been successfully applied previously to the data of the Woods Hole Line W near 39°N and of the RAPID-Scotian line near 42°N (*17*–*19*, *23*).

### Deriving deep western overturning transport relative to and below 1000 m at four latitudes

In this study, both *T*_16_ and *T*_26_ are considered absolute in magnitude, as their respective “eastern endpoints” are defined using a climatological mean. In contrast, the transport values for *T*_39_ and *T*_42_ are relative, with their means arbitrarily set to zero, since they are derived solely from variations at the western boundary.

#### Calculation of T_26_ from the RAPID-MOCHA array near 26.5°N

The methodological approach to deriving the zonally integrated meridional transport profile from the data of the RAPID-MOCHA array, and the corresponding strength of the AMOC near 26.5°N, has been extensively described (*30*, *43*, *44*). Briefly, the approach does not involve measuring directly the pressure gradient on either boundary as in Eq. 2 but rather combining (i) the measurement of the Florida Strait transport by an underwater telecommunication cable, (ii) the calculation of an Ekman transport from zonal wind stresses, and (iii) the measurement of an interior transport from oceanographic moorings. This interior transport is itself obtained by combining direct velocity measurements within a western wedge off the Bahamas archipelago and a geostrophic transport estimated from Eq. 4 using dynamic height endpoint moorings, which are abutting the western wedge to the west and are crawling up the continental slope of Africa to the east. Although there is a slight nonlinearity arising from variations in the depth of the overturning streamfunction maximum, the total AMOC can, to a good approximation, be considered a linear sum of its component contributions (*30*). In addition, the overturning streamfunction calculated by the RAPID project is defined under the constraint of zero net meridional transport across the section. This balance is achieved by introducing a “compensation” term, which is assumed to be a uniform velocity across the entire section (*30*). At the western boundary, Elipot *et al.* (*19*) demonstrated that the western transport profile, obtained by recalculating the transport by holding the eastern boundary dynamic height profile constant for the interior geostrophic transport, was equivalent to the pressure gradient measured directly as a pressure difference between 1382- and 3898-m depths on the western boundary. This result validated the use of Eq. 2 at the RAPID-MOCHA array to estimate meridional transport in that depth range.

In this study, *T*_26_, the deep western overturning transport below and relative to 1000 m is derived exactly as in previous studies (*19*, *23*) as

$$T_{26}=\int_{-4000}^{-1000}\left[-\int_{z}^{-1000}\frac{\partial Q_{W,g}\left(\zeta \right)}{\partial \zeta }\;\mathrm{d\zeta}\right]\;\mathrm{dz}$$ (6)

where *Q*_W,g_(*z*) is a western geostrophic volume transport profile, function of depth *z*, calculated from the RAPID-MOCHA array data by substituting the eastern dynamic height profile and the Ekman transport by their corresponding time-mean values. Suppressing the Ekman transport variability in this calculation ensures that the transport profiles are geostrophic and do not include a component associated with a compensating transport that is enforced in the calculation to maintain a zero net transport across the array at all time steps (*30*).

The uncertainty in the AMOC estimate from the RAPID-MOCHA array (*T*_RAPID_) at 10-day intervals is estimated to be 1.5 Sv (*30*). Since we use the same array data as *T*_RAPID_ to derive *T*_26_, we assign the same uncertainty to this transport time series.

#### Calculation of T_16_ from the MOVE array near 16°N

The MOVE array has been deployed in the western half of the Atlantic basin at 16.5°N since January 2000 (*11*). The data from this array up to October 2022 (access the day via the link in Data, code, and materials availability) are used in this study to derive the time series *T*_16_, as in (*23*). Briefly, the approach consists in deriving a deep western overturning transport by summing the shear of a western interior geostrophic transport from the data of two dynamic height moorings (M1 and M3) following Eq. 4 but with the eastern density profile held constant to its mean value and the shear of a wedge transport from direct velocity measurements acquired by a current meter mooring within the wedge (mooring M4) and current meters on mooring M3, as depicted in fig. S4. Moorings M3 and M4 in the western wedge only directly measure velocities below 1200 m. To address this observational gap, we extended the velocity profiles on both M3 and M4 and western wedge widths by extrapolating the top-most value as a constant at every time step upward from 1200 to 1000 m. For each mooring (M3 and M4), the extrapolated velocity profiles were multiplied by their respective cross-sectional areas to compute individual profiles of transport per unit depth. These two profiles were then summed to obtain the total profile of transport per unit depth in the western wedge. Last, the resulting transport profile was vertically differentiated to derive the western wedge transport shear. The deep western overturning transport *T*_16_ relative to 1000 m, between 1000- and 4000-m depths, is calculated from this combined (western interior and western wedge) shear of transport by integrating twice in depth as in Eq. 6.

The uncertainty in the NADW daily layer transport at the MOVE array (*T*_MOVE_) has been estimated to be 1.4 Sv for the period 2000–2019, attributed to measurement-induced errors (*45*). Given that *T*_16_ and *T*_MOVE_ are derived from the same underlying data, we assume that their uncertainties are comparable. In addition, we also tried to extend the shear of the western wedge transport by extrapolating the top-most value upward as a constant from 1200 to 1000 m at each time step. This adjustment results in only a minor change of 0.1 Sv in the revised *T*_16_.

#### Calculation of T_39_ from the Woods Hole Line W near 39°N

The Woods Hole Line W consisted of five tall moorings (W1 to W5) deployed across the continental slope of the western North Atlantic, between 38°N and 40°N, forming a line nearly perpendicular to the isobaths (*40*), operated from May 2004 to May 2014 (fig. S5) (*25*). Since all the moorings of this array contained near-bottom measurements of ocean velocity and density, as in (*18*, *19*, *23*), we apply the stepping method using Eqs. 2 and 5 to obtain the overturning shear transport in six steps across the continental slope, to calculate *T*_39_, deep western overturning transports, referenced to 1000 m, between 1000- and 4120-m depths, for the entire time period of the Line W array. Further details of the derivation are described next, referencing to the schematic in fig. S5.

The initial step (step 0) calculates the vertical OBP difference (δ*P*) from 1000 m to the base of mooring W0 at 1788 m (this mooring held a bottom pressure recorder instrument from which no data were used in this derivation). Because of the vertical geometry of this step, only density contributes to the pressure gradient. We use data from the McLane Moored Profiler (MMP) instrument on mooring W1 to estimate density at 1000 and 1788 m for the period 2004–2008. For the period 2008–2014, we used data from the MicroCAT Temperature/Conductivity (T/C) recorders at the top and base of mooring W1, interpolated to these depths. Assuming a spatially constant OBP gradient over this step, this one for step 0 is calculated as $\delta P_{0}/\delta z_{0}=-\frac{1}{2}g\left(\rho_{1000m}^{\prime }+\rho_{W0}^{\prime }\right)$ where $\rho_{1000m}^{\prime }$ and $\rho_{W0}^{\prime }$ are the density anomalies at 1000 and 1788 m, respectively, and δ*z*_0_ is the depth difference between 1000 and 1788 m (*17*). The subsequent step (step 1) calculates the OBP gradient between the base of mooring W0 and the base of mooring W1. Data for this calculation come from MMPs, T/C recorders, and Vector Averaging Current Meters (VACMs) deployed at mooring W1. The OBP gradient for step 1 is derived using a modified version of Eq. 5 that uses the averages of the velocity and density anomalies on either side of this step

$$\frac{\delta P_{1}}{\delta z_{1}}=\frac{\bar{\rho }f}{H_{s}}\frac{\left(V_{L,W0}+V_{L,W1}\right)}{2}-g\frac{\rho_{W0}^{\prime }+\rho_{W1}^{\prime }}{2}$$ (7)

where $\bar{\rho }$ is the time mean in situ density between the bases of mooring W0 and W1, *H*_s_ is the slope magnitude between the bases of mooring W0 and W1, *V*_L,W0_ and *V*_L,W1_ are the ocean velocities perpendicular and to the left of the direction from *W*_0_ to *W*_1_, $\rho_{W0}^{\prime }$ and $\rho_{W1}^{\prime }$ are density anomalies at the bases of W0 and W1, respectively, and δ*z*_1_ is the depth difference between the bases of mooring W0 and W1. For steps 2 to 5, the data from other moorings (W2 to W5) and relevant instruments (T/C recorders and VACMs deployed at the bottom of each mooring) are used to compute the OBP gradients in the same manner. Each step assumes a constant OBP gradient within its respective depth range. After determining the OBP gradient for all six discrete steps, we perform a trapezoidal integration, assuming that the OBP at 1000 m is zero, to obtain the deep western overturning transports below and relative to 1000 m at Line W (*T*_39_), from 1000 m to the base of mooring W5 (4120 m). Last, the mean value of *T*_39_, irrelevant for our study, is removed to isolate its temporal anomalies.

#### Calculation of T_42_ from the RAPID-Scotian line near 42°N

RAPID-Scotian line comprised five short moorings (RS1 to RS5) and one tall mooring (RS6), spanning the Scotian continental slope between ~1100- and 3900-m depth, located from 42°N to 42.9°N (fig. S6) (*26*). Each mooring was equipped with an Acoustic Doppler Current Profiler and a MicroCAT temperature and conductivity recorder positioned sufficiently high above the seafloor to estimate the velocity and density of the geostrophic flow (*26*). Using the stepping method, that is, Eqs. 2 and 5, to obtain the overturning shear transport in five steps across the continental slope (from RS1 to RS6) as described in the previous section, we calculate *T*_42_, the deep western overturning transport at RAPID-Scotian line (*T*_42_), referenced to 1100 m, between 1100- and 3900-m depths, for the period of October 2008 to September 2014. Again, we remove the mean value of *T*_42_ for isolating its temporal anomalies.

#### Uncertainty in T_39_ and T_42_

The uncertainties in the transport estimates *T*_39_ and *T*_42_ arise from the uncertainties in the OBP gradient estimates in steps, which, in turn, arise from the uncertainties from density and along-slope velocity measurements (*17*). These uncertainties stem from two primary sources, the instrumental errors and the uncertainties associated with interpolating in time and space various data gaps. In each case, these uncertainties are formally propagated using Eq. 5.

With respect to instrumental errors, the moored instruments deployed at Line W, including MMPs and T/C recorders, have measurement uncertainties of ~0.001°C for temperature and 0.002 for salinity, resulting in a density error of ~0.00015 kg m^−3^. Along-slope velocity measurements from VACMs carry an uncertainty of about 1 cm s^−1^ (*40*). Otherwise, as mentioned in (*18*), the MMP at mooring W1 failed between mid-April 2006 and early April 2007. At mooring W3, the MicroCAT failed between mid-April 2007 and late September 2008. For mooring W4, MicroCAT failures occurred between 2004 and mid-April 2006, as well as between mid-November 2011 and late August 2012. At mooring W5, the MicroCAT experienced a prolonged failure from late March 2008 until the end of the record. To fill these gaps in the density records, we synthesized data using regressions based on data from the adjacent moorings during other time periods. For the velocity records, the near-bottom current meter at mooring W1 failed on 6 December 2004, resulting in a data gap until 30 April 2005 (*18*). Instead, we used near-bottom velocity data recorded by the MMP at mooring W1, interpolated to daily values. At mooring W4, the VACM at the bottom failed during the 2004–2006 deployment. We instead used data from the VACM positioned 452 m above the bottom (*18*). At mooring W4, the current meter also failed between early November 2011 and late August 2012. The final velocity gap occurred between early February 2008 and early June 2009 at mooring W5. To fill the last two gaps, we applied depth-based linear extrapolation models using data from three adjacent moorings during the same time. We quantify the uncertainties introduced by the application of regression and extrapolation models using the root mean squared error (RMSE). Last, the combined uncertainties, including instrumental errors and interpolation uncertainties, define the total uncertainty in the OBP difference at each step. We perform a trapezoidal integration of the uncertainties in the OBP difference across all discrete steps to yield a time-varying uncertainty in *T*_39_, ranging from 1.71 to 4.62 Sv, with a mean uncertainty of 2.17 Sv.

A previous study estimated that the uncertainty in *T*_42_, derived using the stepping method, was 0.82 Sv for the period from October 2008 to September 2009, taking into account instrumental errors as well as the effect of sampling and representation errors (*17*). In our study, we adopt this uncertainty value for the entire period under investigation. In addition, we used depth-based linear extrapolation models to fill three multimonth density gaps at Moorings RS4, RS5, and RS6, occurring in 2010 and 2011, based on density data from RS1 to RS3 (*26*). We also used depth-based linear extrapolation models to fill velocity gaps: from late September 2011 to early March 2013 at RS2, from the beginning of the record to mid-December 2010 at RS5, and from mid-December 2010 to early October 2011 at RS6. When incorporating the uncertainties derived from the RMSE of these extrapolation models, the maximum uncertainty in our estimated *T*_42_ increases to 1.25 Sv.

### Statistical methods

#### Trend estimations

Linear trends and their uncertainties are estimated using the methodology proposed in (*46*), implemented in the Hector software package available at https://teromovigo.com/product/hector/. This approach identifies linear trends within time series affected by correlated noise and potential seasonal cycles, simultaneously estimating the trend and model parameters for both noise and seasonality. Maximum likelihood estimation is used to determine all parameters and their uncertainties. While trend values obtained through this method are nearly identical to those obtained using ordinary least squares, their uncertainties are often underestimated because of the presence of correlated noise, a characteristic that can be inferred by analyzing the spectrum estimates. This analysis leads to the choice of a Matèrn process model (*47*) for the noise of each time series, which is equivalent to selecting a model for the spectrum estimates. A general Gauss-Markov model was also considered, but the Matèrn model was selected on the basis of the Akaike information criteria (AIC) (*48*). The decision to include or exclude a seasonal model consisting of one harmonic at the annual frequency, one harmonic at the semiannual frequency or both, is also made based on the AIC. A seasonal cycle with both annual and semiannual components is justified for *T*_26_ and *T*_42_. A seasonal cycle with a single annual component is found to be appropriate for *T*_RAPID_E_, whereas a seasonal cycle incorporating a semiannual component is justified for *T*_RAPID_ and *T*_RAPID_W_. Reported uncertainties for trend estimates ultimately correspond here to two SEs.

#### Spectral and cross-spectral estimations

Auto-[*S_xx_*(*f*)] and cross-spectral estimates [*S_xx_*(*f*)] are obtained by the multitaper method, choosing a time-bandwidth product of 3 and 5 Slepian tapers (*49*). To investigate linear relationships between two time series as a function of frequency, we calculate the coherence squared and the coherence phase from the autospectral and cross-spectral estimates as $\frac{{|S_{\mathrm{xy}}|}^{2}\left(f\right)}{S_{\mathrm{xy}}\left(f\right)\;S_{\mathrm{xy}}\left(f\right)}$ and $\text{arg}\left[S_{\mathrm{xy}}\left(f\right)\right]$, respectively. Linear trends are removed from time series before estimating spectral quantities.
