# Supplementary material for: Meridionally consistent decline in the observed western boundary contribution to the Atlantic Meridional Overturning Circulation
Source: Sci Adv. 2026 Apr 8;12(15):eadz7738. doi: 10.1126/sciadv.adz7738 (PMC13060603; doi:10.1126/sciadv.adz7738)
Supplement: Supplementary file 1 — Figs. S1 to S6 Table S1 [file sciadv.adz7738_sm.pdf]

Science Advances
AAAS

Supplementary Materials for

Meridionally consistent decline in the observed western boundary contribution to the Atlantic Meridional Overturning Circulation

Qianjiang Xing *et al.*

Corresponding author: Qianjiang Xing, bryant5231314@gmail.com

Sci. Adv. **12**, eadz7738 (2026)
DOI: 10.1126/sciadv.adz7738

This PDF file includes:

Figs. S1 to S6
Table S1

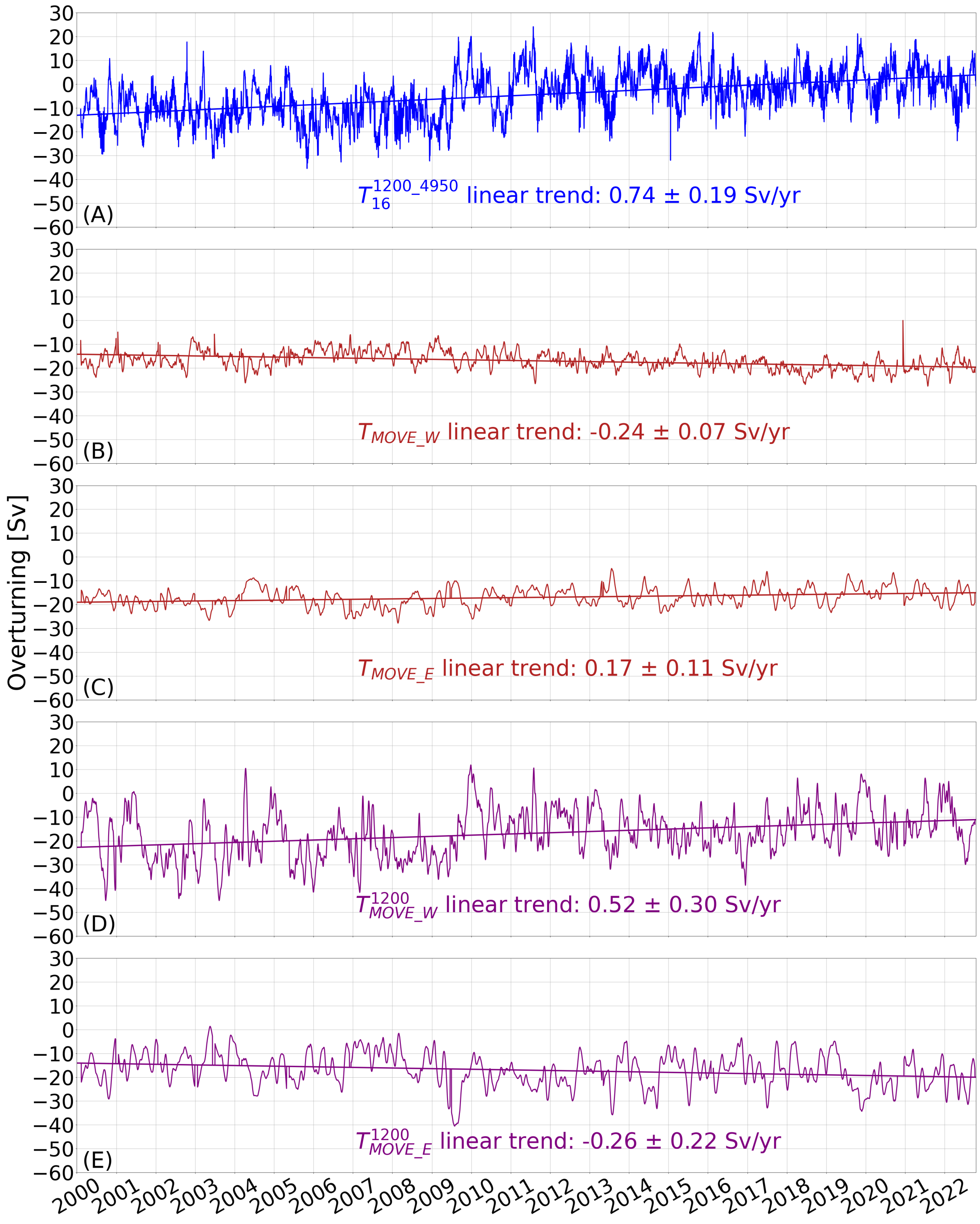

Overturning [Sv]
$T_{16}^{1200\_4950}$ linear trend: 0.74 ± 0.19 Sv/yr
(A)
T_{MOVE_W} linear trend: -0.24 ± 0.07 Sv/yr
(B)
T_{MOVE_E} linear trend: 0.17 ± 0.11 Sv/yr
(C)
$T_{MOVE\_W}^{1200}$ linear trend: 0.52 ± 0.30 Sv/yr
(D)
$T_{MOVE\_E}^{1200}$ linear trend: -0.26 ± 0.22 Sv/yr
(E)
30
20
10
0
−10
−20
−30
−40
−50
−60
2000
2001
2002
2003
2004
2005
2006
2007
2008
2009
2010
2011
2012
2013
2014
2015
2016
2017
2018
2019
2020
2021
2022

Figure S1: **Alternative derivations of deep western overturning and NADW layer transports at the MOVE array.** (A) $T_{16}^{1200\_4950}$: deep western overturning transport between 1200 m and 4950 m, relative to 1200 m. (B) T_{MOVE_W}: NADW layer transport recalculated by holding the eastern dynamic height (from mooring M1) as constant over time. (C) T_{MOVE_E}: NADW layer transport recalculated by holding the western dynamic height and wedge transport (from moorings M3 and M4) as constant over time. (D) $T_{MOVE\_W}^{1200}$: NADW layer transport recalculated by holding the eastern dynamic height as constant over time and using a 1200-m reference level. (E) $T_{MOVE\_E}^{1200}$: NADW layer transport recalculated by holding the western dynamic height and wedge transport as constant over time and using a 1200-m reference level. Linear trend estimates are displayed as solid lines and their values and uncertainties are indicated below each curve.

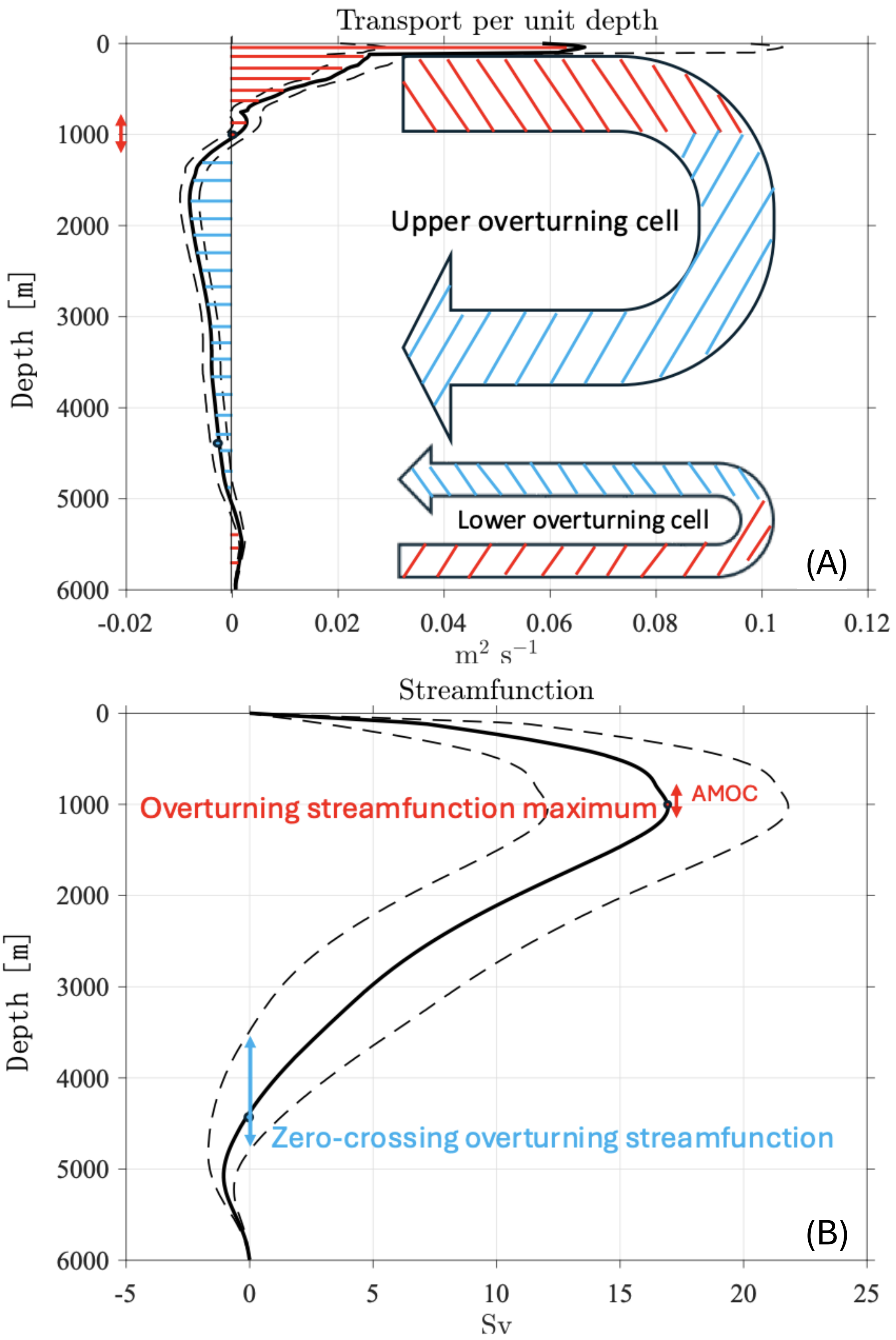

Transport per unit depth
Upper overturning cell
Lower overturning cell
(A)
Depth [m]
m² s⁻¹
0
1000
2000
3000
4000
5000
6000
-0.02
0
0.02
0.04
0.06
0.08
0.1
0.12
Streamfunction
Overturning streamfunction maximum
AMOC
Zero-crossing overturning streamfunction
(B)
Depth [m]
Sv
0
1000
2000
3000
4000
5000
6000
-5
0
5
10
15
20
25

Figure S2: **Overturning cells and streamfunction at RAPID-MOCHA array.** (A) Transport per unit depth (m^2 s^{-1}) derived from RAPID-MOCHA observations spanning 2004–2023. The solid black line represents the time-mean and the black dashed curves indicate plus or minus one standard deviation around the time-mean. The red hatching indicates northward transport, whereas blue hatching represents southward transport. (B) Overturning streamfunction (Sv) over the period 2004–2023. The solid black line represents the time-mean and the black dashed curves indicates plus or minus one standard deviation around the time-mean. The red double-headed arrow indicates the depth range of the overturning streamfunction maximum (600–1200 m). The time-varying maximum of the overturning streamfunction defines the Atlantic Meridional Overturning Circulation (AMOC) strength at each time step. The blue double-headed arrow indicates the depth range of the zero-crossing of the overturning streamfunction (3500–4800 m). In panel (A), the sum of the integral of the northward transport per unit depth (red) above the depth of the streamfunction maximum, and the integral of the southward transport per unit depth from that depth to the zero-crossing depth of the streamfunction, sum to zero, thereby forming the upper overturning cell at the 26.5°N. Similarly, the integrals of the northward and southward transport per unit depth below the depth of the zero-crossing overturning streamfunction sum to zero and constitute the lower overturning cell.

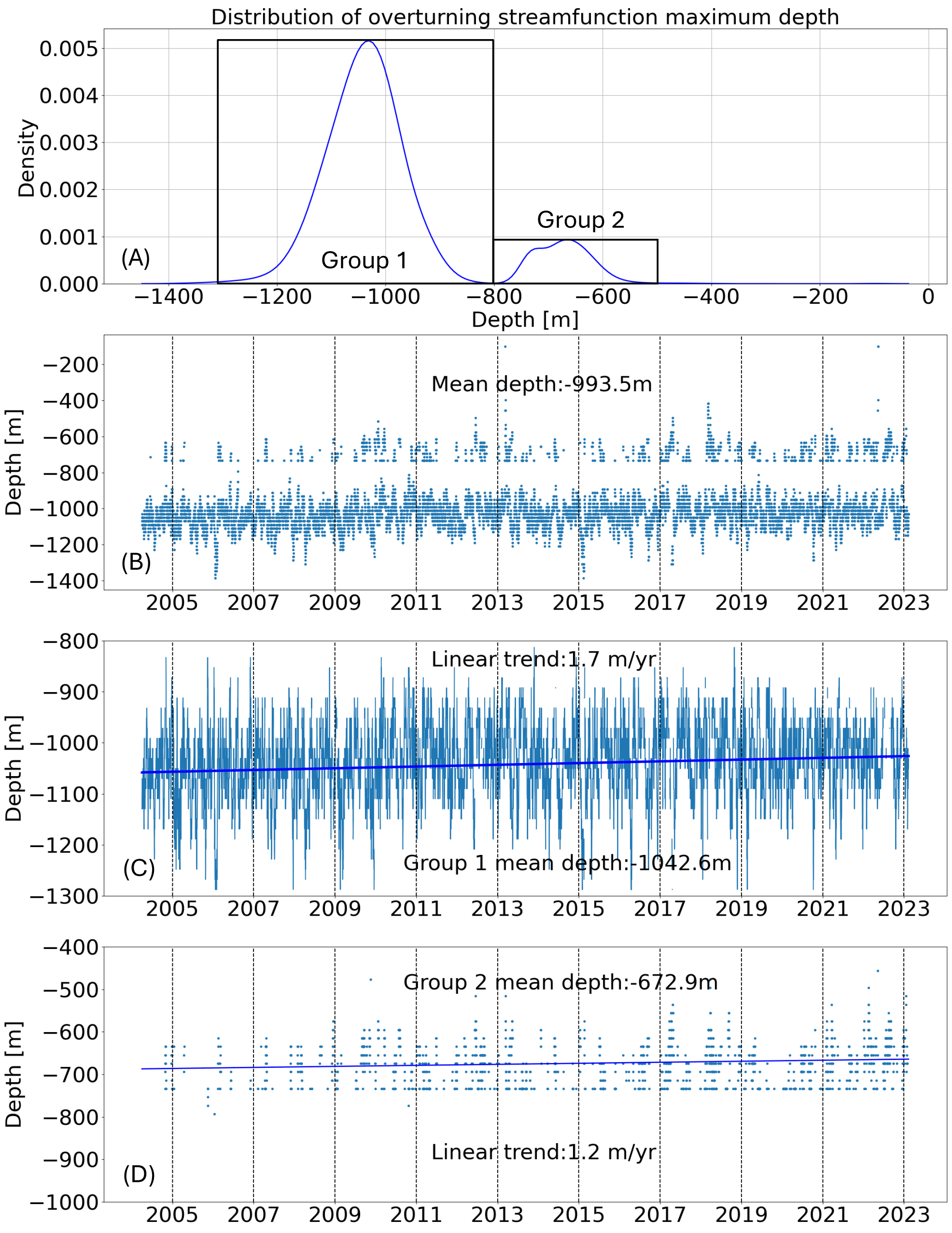

Figure S3: **The depth of the overturning streamfunction maximum at 26.5°N from RAPID-MOCHA observations for the period 2004–2023 is not temporally constant.** (A) Distribution of the depth of the overturning streamfunction maximum. (B) Time series of the depth of overturning streamfunction maximum. (C) Time series for the data of Group 1 (800-1300 m depth range) of the distribution as delineated in panel (A). (D) Time series for the data of Group 2 (shallower than 800 m). The time-mean and trend values of the time series are indicated in each panel.

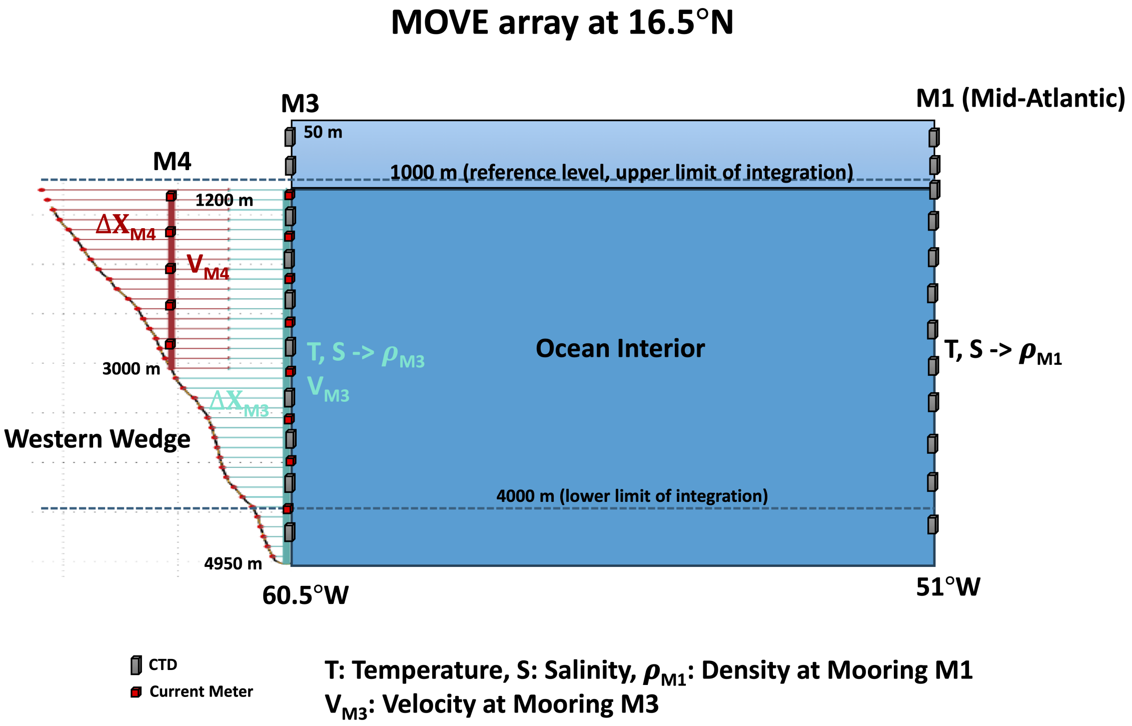

Figure S4: **Schematic of the Meridional Overturning Variability Experiment (MOVE) array at 16.5°N in the Atlantic Ocean.** This array consists of three moorings designed to monitor oceanic density and velocity profiles. M1 is a tall mooring located at the Mid-Atlantic Ridge, equipped with temperature and salinity sensors to derive density profiles. M3, another tall mooring situated at the western boundary, is outfitted with both temperature and salinity sensors and current meters, providing density and velocity profiles. Together, M1 and M3 act as vertical boundaries delineating the ocean interior of the western half of the Atlantic basin at 16.5°N. Density profiles between 50 m to 4950 m from M1 and M3 moorings are used to derive the shear of the ocean interior geostrophic volume transport. The western wedge of the array is formed of a continental slope to the west and M3 to the east, with a short mooring M4 positioned mid-wedge. Mooring M4 is equipped solely with current meters from which are derived velocity profiles between 1200 m and 3000 m. Note that M3 generates velocity profiles only between 1200 to 4950 m. The horizontal distances ΔX_{M3} and ΔX_{M4} can be used to calculate the cross-sectional areas of the western wedge. The depths 1000 m and 4000 m are upper and lower limits of integration for calculating the deep western overturning transport at 16.5°N. Reference level is also chosen at 1000 m.

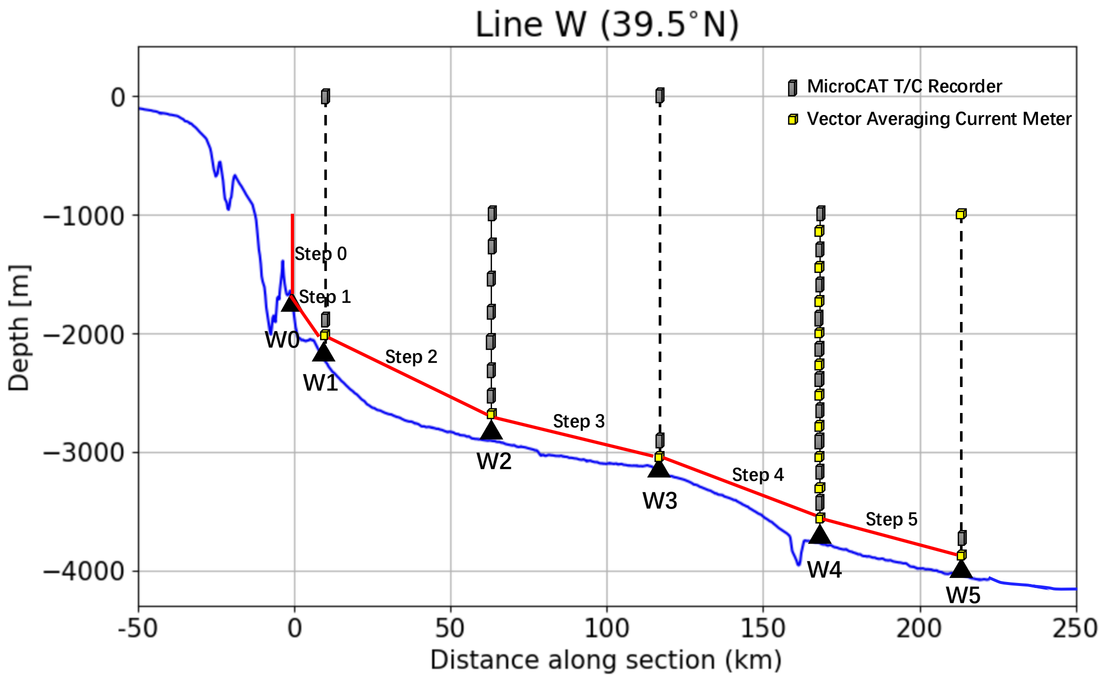

Figure S5: **Schematic of the moorings constituting the Woods Hole Oceanographic Institution Line W.** The vertical-dashed lines at sites W1, W3, and W5 are moorings equipped with McLane profilers. Grey rectangles represent temperature and salinity measuring instruments (MicroCAT T/C recorders). Yellow cubes represent direct velocity measuring instruments (Vector Averaging Current Meters). Black triangles represent bottom pressure recorders (their data were not used in this study). The red segments indicate the steps between moorings when using the "stepping method" with density and velocity records to calculate the deep western overturning transport at 39.5°N.

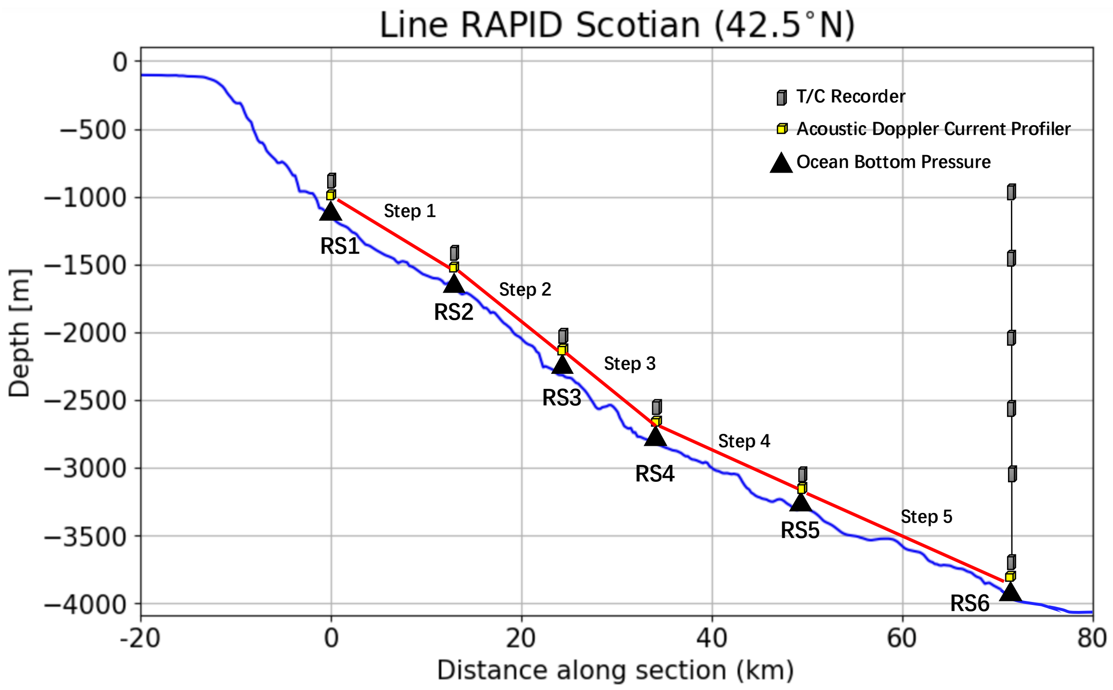

Figure S6: **Schematic of the moorings constituting the RAPID-Scotian line.** Grey rectangles indicate temperature and salinity measuring instruments (MicroCAT T/C recorders). Yellow cubes indicate direct velocity measuring instruments, Acoustic Doppler Current Profilers (ADCPs). T/C recorders and ADCPs are located at the bottom of moorings RS1 to RS6. Mooring RS6 additionally holds T/C recorders at several depths. The red segments indicate the steps between moorings when using the "stepping method" to calculate deep western overturning transport at 42.5°N.

Table S1: **Linear temporal trend [Sv/yr] for T_{16}, T_{26}, T_{39}, and T_{42} during different periods, estimated by modeling mixed effects.** Each column corresponds to different overlapping periods indicated as year/month/day. The column "All" lists the trend estimates for the entire length of each individual time series. Uncertainties are 2-σ confidence intervals.

Transport	All	2008/10/03-2014/05/02	2004/05/02-2014/05/02	2004/04/02-2022/10/18
T_{16}	0.67 ± 0.13	1.93 ± 1.42	1.22 ± 0.48	0.70 ± 0.20
T_{26}	0.26 ± 0.07	0.18 ± 0.45	0.53 ± 0.20	0.26 ± 0.07
T_{39}	0.45 ± 0.17	-0.11 ± 0.38	0.44 ± 0.17	
T_{42}	0.10 ± 0.17	0.08 ± 0.18		
